# Influence of Polymerization Protocol on Adhesion and Proliferation of *Streptococcus mutans* on Three Dental Composite Resins

**DOI:** 10.3390/biomedicines12102235

**Published:** 2024-10-01

**Authors:** Francesco De Angelis, Camillo D’Arcangelo, Silvia Di Lodovico, Edoardo Sorrentino, Matteo Buonvivere, Simonetta D’Ercole

**Affiliations:** 1 Department of Medical, Oral and Biotechnological Sciences, “G. d’Annunzio” University of Chieti–Pescara, Via dei Vestini 31, 66100 Chieti, Italy; camillo.darcangelo@unich.it (C.D.); sorrentinoed@libero.it (E.S.); m.buonvivere@gmail.com (M.B.); simonetta.dercole@unich.it (S.D.); 2 Department of Pharmacy, “G. d’Annunzio” University of Chieti–Pescara, Via dei Vestini 31, 66100 Chieti, Italy; silvia.dilodovico@unich.it

**Keywords:** *Streptococcus mutans*, composite resins, light-cured, heat-cured, polymerization

## Abstract

**Background/Objectives:** The aim of this in vitro study was to analyze and compare the *Streptococcus mutans* ability to adhere and form biofilm on the surface of light-cured VS heat-cured dental composite resins; **Methods:** Three composite resins with different chemical formulations were selected: GrandioSO (GR), Venus Diamond (VD) and Enamel Plus Hri Biofunction (BF). Disk-shaped specimens were manufactured by light-curing the composite resins (light-cured subgroups) and subjecting them to a further heat-curing cycle at 80° for 10 min (heat-cured subgroups). Specimens were analyzed for planktonic CFU count (CFU/mL), sessile CFU count (CFU/mL) and for biomass quantification (OD_570nm_); **Results:** The planktonic CFU count was higher in all the light-cured subgroups than in the heat-cured subgroups (light-cured: GR = 7.23 × 10^6^, VD = 2.14 × 10^7^, BF = 4.40 × 10^7^; heat-cured: GR = 4.89 × 10^6^, VD = 4.95 × 10^6^, BF = 2.80 × 10^7^), with a statistically significant increase for BF and VD. Focusing on the sessile CFUs, both GR (light-cured = 7.49 × 10^6^; heat-cured = 3.97 × 10^6^) and VD (light-cured = 2.93 × 10^7^; heat-cured = 6.07 × 10^6^) showed a significantly increased number of colonies in the light-cured subgroups. The OD_570nm_ values recorded for the light-cured BF subgroup (0.4280) were significantly increased compared to the heat-cured BF subgroup (0.1931); **Conclusions:** A more complete polymerization protocol seems to lead to a potential reduction in the risk of secondary caries.

## 1. Introduction

Dental decay is a disease with multifactorial etiology. It is caused by cariogenic bacteria in the presence of fermentable carbohydrates, but is also influenced by external factors and individual susceptibility. The Gram-positive *Streptococcus mutans* is one of the main cariogenic bacteria. It survives at low pH and its pathogenesis is related to the production of organic acids following carbohydrate metabolism [1,2]. *Streptococcus mutans* acid release seems to act even on dental composites, thus predisposing pre-existing restorations to the risk of secondary caries [3,4]. The longevity of the restoration can also be affected by the shrinkage of the composite during polymerization, which may promote the occurrence of tooth decay [5]. Depending on the more or less virulent microbial strain, the esterase activity of *Streptococcus mutans* can deteriorate [6] and modify the surface morphology of composite resins [7]. Also, it may inhibit the activity of glycolysis-related enzymes [8]. *Streptococcus mutans* is able to form biofilm on solid surfaces [9,10,11,12] following glucosyltransferases (GTFs) production to catalyze extracellular polysaccharides synthesis (EPS) [13,14]. Its ability to form biofilm is greater than many other Streptococcus spp. that populate the oral cavity environment [15,16]. Some specific adhesins of *Streptococcus mutans*, known as dual antigen I/II, promote biofilm formation, mediating bacterial attachment to the tooth’s salivary pellicle [17,18,19] and interacting with other bacteria and host proteins [17,18,19,20]. Biofilm roughens and degrades the surfaces of composite resins [7], promoting bacterial colonization at the tooth–composite interface [21] and resulting in secondary caries [22] and pulpal disease [23]. Surface roughness (Ra) and surface free energy can affect bacterial adhesion [24]. A Ra greater than 0.2 µm is associated with greater bacterial colonization [25]. Lifshitz–Van der Waals forces, acid-base and electrostatic interactions generally affect bacterial adhesion to surfaces [26]. According to the DLVO (Derjaguin–Landau–Verweij e Overbeek) theory, these forces can be combined [27]. 

A polymerization reaction is defined as the degree of conversion of monomers into polymers. Direct resin composite restorations are light-cured chairside for 20–40 s, while indirect restorations typically undergo additional extra-oral heat-curing cycles before an effective intra-oral adhesive cementation [28,29,30]. Light-curing reactions never guarantee total monomeric conversion [31]; in fact, up to 45% of unreacted monomers may remain unpolymerized [32]. A heat-curing procedure promotes greater, even if not complete, monomer conversion into polymer chains [33,34], enhancing properties such as microhardness, flexural strength, fracture toughness, wear resistance, tensile strength and color stability [35,36,37,38].

Heat-curing seems also to reduce monomer leachability into saliva [39], which appears as relevant since previous research suggested that the release of ethylene glycol-dimethyl acrylate (EGDMA) and triethylene glycol-dimethacrylate (Teg-GDMA) from composite resins might supposedly enhance the growth of some *Streptococcus* spp. [40]. It has been observed that the light-curing time may affect bacteria surface colonization on resin composites [41]. For Kraigsley and coll. [42], the degree of conversion of cross-linked dimethacrylate polymers alters the biofilm metabolic activity. Furthermore, according to D’Ercole and coll. [43], in vitro bacterial adhesion and proliferation can be affected by the composite composition. Despite the well-known improvements in mechanical properties achieved following heat-curing protocols, studies concerning their effects on bacterial adhesion and proliferation are still lacking. Thus, the aim of this preliminary in vitro study was to analyze and compare the ability of *Streptococcus mutans* to adhere, proliferate and form biofilm on the surface of three commercially and nanohybrid available light- and heat-cured dental composite resins (GrandioSO: GR; Venus Diamond: VD; Enamel Plus Hri Biofunction: BF), each having different chemical formulations. These particular materials were selected because they had been already tested in presence of another common commensal cariogenic agent [44]. The null hypothesis was that no difference in terms of antibacterial and antibiofilm properties among chemically different and differently cured resin composites could be detected.

## 2. Materials and Methods

A summary of the experimental groups and subgroups, together with a list of the materials included in the present experimental design, is given in Table 1.

### 2.1. Realization of Composite Disks

Disk-shaped specimens were manufactured by placing uncured composite resin in polyvinylsiloxane molds (2 × 4 mm). The filled molds were positioned between two glass slides held in place with a paper clip to extrude the surplus material. As summarized in Table 1, for light-cured subgroups, a light-emitting diode curing unit (Celalux 3, VOCO, Cuxhaven, Germany) with an 8 mm tip diameter and an output power of 1300 mW/cm^2^ was used to cure each disk for 20 s from the top surface and 20 s from the bottom surface [43]. Each composite disk had a total surface area of 50.27 mm^2^. All disks were washed in an ultrasonic bath with distilled water for 10 min. 

For heat-cured subgroups, disk-shaped specimens were made following the same protocol described for light-cured subgroups. Moreover, following the ultrasonic bath, samples received additional heat-polymerization in a composite heat-curing unit at 80 °C for 10 min (LaborLux, Micerium, Avegno, Genova, Italy).

The sample size for the present research was established, taking into account previous similar studies [43,44]. On this basis, a total of 339 light-cured and 339 heat-cured composite disks were manufactured for each different resin composite under investigation and used for the subsequent experimental steps.

### 2.2. Saliva Collection

According to a previously described protocol, human saliva samples were taken from healthy volunteers with an age > 18 years. The Ethics Committee of University “G. d’Annunzio”, Chieti–Pescara, Italy (approval code SALI, N. 19 of the 10 September 2020), approved the collection and the use of saliva [45].

Saliva was pooled, mixed, centrifuged (16.000× *g* for 1 h at 4 °C) and filtered from microorganisms (filters diameters: 0.8 μm, 0.45 μm, and 0.2 μm). To consider saliva samples sterile, no detected bacterial growth in both aerobic and anaerobic atmospheres after incubation for 48 h at 37 °C is required [45]. Sterile saliva was collected and kept frozen in sterile tubes until the study was carried out.

### 2.3. Bacterial Strain

The clinical bacterial strain *Streptococcus mutans* CH02 was isolated from the oral cavity of patients at the dental clinic of the University “G. d’Annunzio”, Chieti–Pescara [43]. The frozen (−80 °C) strain was recovered in Brain Heart Infusion broth (BHI, Oxoid, Milan, Italy) overnight at 37 °C under an anaerobic condition. The broth culture was diluted 1:10 in BHI broth containing 1% (*w*/*v*) sucrose and refreshed for 2 h at 37 °C in a shaking thermostatic water bath (120 rpm). Bacterial suspension was prepared using a spectrophotometer (Eppendorf) to obtain an optical density of OD_600_ = 0.125 corresponding to 9 × 10^6^ CFU/mL. 

All composite disk specimens were placed in 96-well polystyrene microtiter plates and the top and bottom surfaces were sterilized through the action of ultraviolet UV lights for 30 min. 

To form the protein pellicle layer on the surface, sterile specimens were inoculated for 2 h in saliva at 37 °C in a shaking incubator. Biofilms were grown on each composite disk by inoculating 200 µL broth culture of *Streptococcus mutans* CH02 and incubating at 37 °C for 24 h under anaerobic conditions, and then for another 24 h in an aerobic atmosphere. As a negative control, salivary film-coated disks were incubated in bacteria-free culture media to confirm the sterility of the disks and the adsorption of the biomass dye.

After incubation, microbial growth was quantitatively analyzed for the following aspects:

(I) Planktonic CFU count of the bacterial cells (CFU/mL);
(II) Sessile CFU count of the cultivable cells on composite disks (CFU/mL);
(III) Biomass evaluation of the biofilm produced on composite disks using Hucker’s crystal violet staining method (OD_570nm_).

Each quantitative test was performed in triplicate for three independent experiments.

Moreover, representative specimens from each subgroup were qualitatively analyzed to assess the biofilm morphology via Scanning Electron Microscope (SEM) evaluation.

### 2.4. Planktonic CFU Count

For the planktonic CFU count, the planktonic bacterial phase removed from the wells containing the resin disks was vortexed, diluted in PBS and spread on Tryptic Soy Agar (TSA) plates. Then, it was incubated in anaerobic conditions for 48 h at 37 °C. The CFU/mL count was performed. For the detection, 9 disks for the light-cured and 9 disks for the heat-cured subgroups of each composite resin material in triplicate were analyzed (9 light-cured GR and 9 heat-cured GR disks, 9 light-cured VD and 9 heat-cured VD disks, 9 light-cured BF and 9 heat-cured BF disks) for a total of 81 light-cured and 81 heat-cured disks.

### 2.5. Sessile CFU Count

The disks were placed in a sterile test tube containing 1 mL PBS. Then, each test tube was placed in a 4 kHz ultrasonic water bath (Euronda, Vicenza, Italy) for 3 min, followed by vortex mixing for 2 min to detach the *Streptococcus mutans* CH02 adhering to the surface of each disk. The live/dead analysis was carried out to evaluate the non-negative effect of the sonication and the vortex. The samples were diluted with PBS and spread on TSA plates. Finally, they were incubated for 48 h under anaerobic conditions at 37 °C, followed by the counting of CFU/mL. For this detection, 9 disks for the light-cured and 9 disks for the heat-cured subgroups of each composite resin material in triplicate were analyzed (9 light-cured GR and 9 heat-cured GR disks, 9 light-cured VD and 9 heat-cured VD disks, 9 light-cured BF and 9 heat-cured BF disks) for a total of 81 light-cured and 81 heat-cured disks.

### 2.6. Biomass Quantification by Optical Density (OD_570nm_)

Crystal-violet (CV) staining [44,46,47,48] was used to evaluate relative biofilm biomass formed by *Streptococcus mutans* CH02 on composite surfaces after 48 h for biofilm formation. The disks were washed three times with PBS, left to air dry, stained with crystal-violet 0.1% (Sigma–Aldrich, Milan, Italy) for 1 min and washed with PBS. Then, the CV was eluted with ethanol and the biofilm formation was quantified by measuring absorbance at 570 nm with a microplate reader (SAFAS, Munich, Germany). For this detection, 18 disks for each light-cured and 18 disks for each heat-cured subgroup were analyzed in triplicate, including negative controls (9 light-cured GR and 9 light-cured GR negative controls, 9 heat-cured GR and 9 heat-cured GR negative controls, 9 light-cured VD and 9 light-cured VD negative controls, 9 heat-cured VD and 9 heat-cured VD negative controls, 9 light-cured BF and 9 light-cured BF negative controls, 9 heat-cured BF and 9 heat-cured BF negative controls), for a total of 162 light-cured and 162 heat-cured disks.

### 2.7. Scanning Electron Microscope (SEM) Analysis

After 48 h of in vitro biofilm formation, representative specimens from each subgroup were fixed for 1 h in 2.5% glutaraldehyde, dehydrated in six ethanol washes (10%, 25%, 50%, 75% and 90% for 20 min and 100% for 1 h), and dried overnight in a bacteriological incubator at 37 °C. Then, they were observed using a SEM (EVO 50 XVP LaB6, Carl Zeiss SMT Ltd., Oberkochen, Germany) at 15 kV, under 500×, 1000× and 2000× magnifications after gold-sputter coating (Emitech K550, Emitech Ltd., Montigny Le Bretonneu, France). A total of 5 composite disks for each light-cured subgroup and 5 disks for each heat-cured subgroup were addressed via SEM analysis.

### 2.8. Statistical Analysis

Means and standard deviations for data coming from the quantitative experiments (planktonic CFU count, sessile CFU count, biomass quantification by OD_570nm_) were calculated in each subgroup. Statistical analysis was performed using SPSS for Windows version 21 (IBM SPSS Inc., Armonk, NY, USA), by means of Two-Way Analysis of Variance (ANOVA) and Tukey tests for post hoc intergroup comparisons. *p*-values less than 0.05 were considered significant.

## 3. Results

The detailed results (means and standard deviations) of the quantitative tests performed on the three composites that were analyzed and polymerized according to the two curing protocols under investigation are summarized in Table 2.

The planktonic CFU counts recorded for BF (light-cured = 4.40 × 10^7^ CFU/mL; heat-cured = 2.80 × 10^7^ CFU/mL) were significantly higher than those obtained for GR (light-cured = 7.23 × 10^6^ CFU/mL; heat-cured = 4.89 × 10^6^ CFU/mL) and VD (light-cured = 2.14 × 10^7^ CFU/mL; heat-cured: 4.95 × 10^6^ CFU/mL), concerning both curing protocols. No statistically significant differences were recorded between the heat-cured GR subgroup and the heat-cured VD subgroup (*p* < 0.05).

The sessile CFU counts recorded on both GR (light-cured = 7.49 × 10^6^ CFU/mL; heat-cured = 3.97 × 10^6^ CFU/mL) and VD (light-cured = 2.93 × 10^7^ CFU/mL; heat-cured = 6.07 × 10^6^ CFU/mL) were significantly higher in the light-cured subgroups compared to the heat-cured subgroups. No significant differences were recorded between the light-cured (6.71 × 10^6^ CFU/mL) and heat-cured (6.38 × 10^6^ CFU/mL) BF subgroups (*p* < 0.05). Specifically, the light-cured VD subgroup had the highest CFU count for the sessile phase cells ever. 

The biomass quantification by optical density recorded in the light-cured BF subgroup (0.4280 OD_570nm_) was significantly higher than for the same heat-cured material (0.1931 OD_570nm_) (*p* < 0.05). No significant differences were recorded between the light-cured (0.1325 OD_570nm_) and the heat-cured (0.1464 OD_570nm_) GR subgroups and between the light-cured (0.1457 OD_570nm_) and the heat-cured (0.1731 OD_570nm_) VD subgroups.

**Figure 1:**
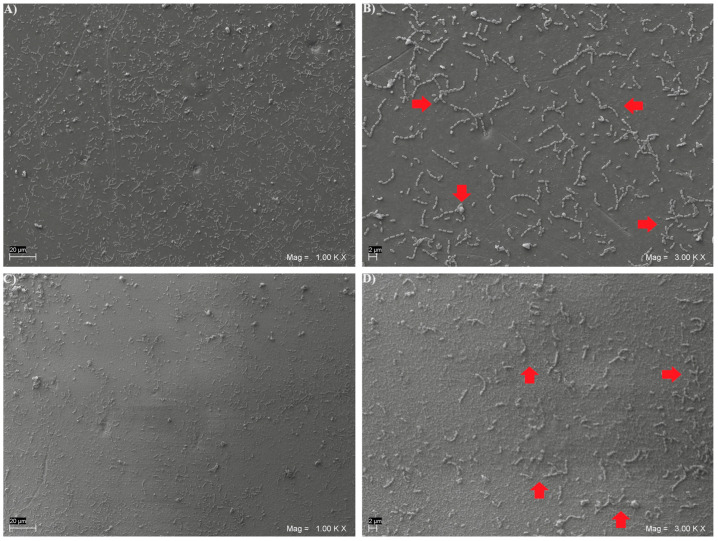
Representative SEM microphotographs of *Streptococcus mutans* colonies formed on disk-shaped specimens from light-cured GR subgroup, at 1000× (**A**) and 3000× (**B**) magnifications, and from heat-cured GR subgroup, at 1000× (**C**) and 3000× (**D**) magnifications. Arrows in 3000× SEM images indicate examples of *Streptococcus mutans* colonies.

**Figure 2:**
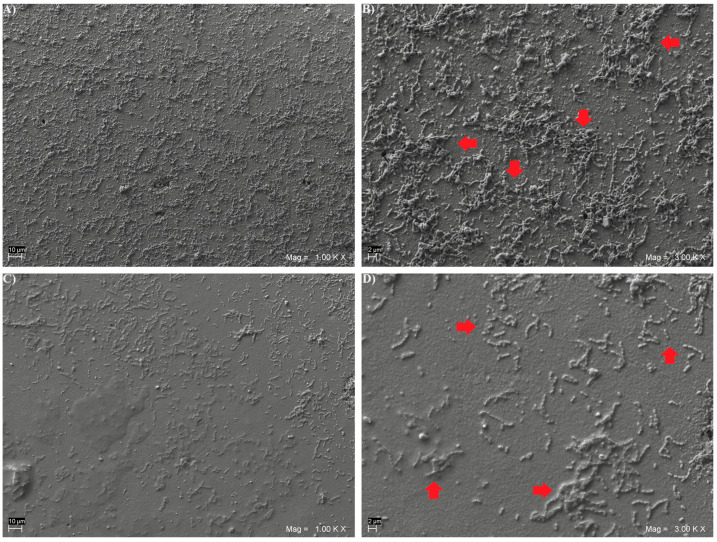
Representative SEM microphotographs of *Streptococcus mutans* colonies formed on disk-shaped specimens from light-cured VD subgroup, at 1000× (**A**) and 3000× (**B**) magnifications, and from heat-cured VD subgroup, at 1000× (**C**) and 3000× (**D**) magnifications. Arrows in 3000× SEM images indicate examples of *Streptococcus mutans* colonies.

**Figure 3:**
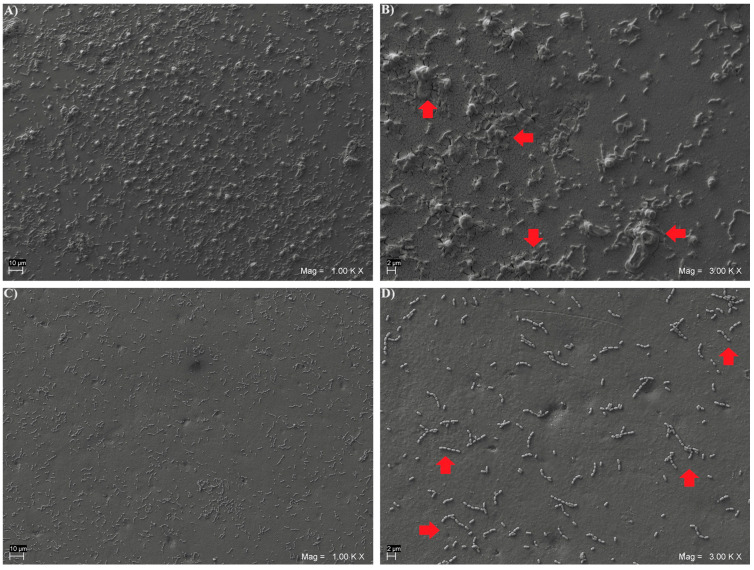
Representative SEM microphotographs of *Streptococcus mutans* colonies formed on disk-shaped specimens from light-cured BF subgroup, at 1000× (**A**) and 3000× (**B**) magnifications, and from heat-cured BF subgroup, at 1000× (**C**) and 3000× (**D**) magnifications. Arrows in 3000× SEM images indicate examples of *Streptococcus mutans* colonies.

SEM microphotographs, taken at 1000× and 3000× magnifications, showing the *Streptococcus mutans* CH02 cells adherent on the surface of the three light and heat-cured composite resins, are given in Figure 1, Figure 2 and Figure 3. An increased number of sessile colonies were observed in the light-cured subgroups. 

## 4. Discussion

The null hypothesis tested in the present study had to be rejected. In the present study, significant differences in terms of bacterial growth and biofilm formation in the presence of chemically different and differently cured resin composites were recorded. Both the specific composite resins composition and the polymerization protocol proved to be relevant factors, able to significantly influence the behavior of *Streptococcus mutans*.

In the present study, the three composite resins tested (Table 1) were selected because they are well-known and widely used nanohybrid dental composites that significantly differ one another in terms of formulation, both concerning the resin matrix chemicals and the filler load, size and composition. Furthermore, previous research [44] has recently analyzed the effect of these three resin composites on the adhesion and proliferation of a common commensal cariogenic agent, *Candida albicans* [49,50,51,52], making it relevant to further investigate the behavior of the main cariogenic bacteria, *Streptococcus mutans*, in the presence of the same materials. 

Specific characteristics of each different material, surface properties in particular, may influence bacterial adhesion [25]. A reduced organic matrix microhardness favors inorganic fillers exposure following material wear by attrition [53]. Nanometric-sized composite fillers lead to superior esthetic characteristics due to their reduced dimensions and better distribution [54,55]. Also, the amount, shape and size of the filler can affect the bacterial adhesion to the composites surface [53,54,56]. A larger filler dimension promotes greater roughness [55,57,58,59,60]. Smooth surfaces show a reduced bacterial adherence and accumulation than rough surfaces [59], retarding biofilm adherence and growth [61]. In the present study, the small size of the micrometric filler in GR (max 1 µm) and BF (max 3 µm) composites compared to the VD composite (max 20 µm) could hypothetically justify the increased sessile proliferation of *Streptococcus mutans* CH02 on the latter material. In order to limit surface differences, in the present protocol, all composite disks were light-cured within polyvinylsiloxane molds and between two glass slides, which guaranteed standardized and predictably smooth surfaces, with no need of any final polishing steps. Consequently, it was possible to exclude the potential confounding effect of any difference in surface roughness among hand-polished materials, focusing the attention just on the two variables under investigations (i.e., curing protocol and composite formulation). Moreover, the use of molds and glass slides allowed to prevent the composite material from coming into contact with oxygen, avoiding the formation of any oxygen inhibition layer (OIL) on the composite surface. OIL may negatively affect the polymerization reaction, reducing the monomeric degree conversion [62,63,64,65,66]. Additionally, the tested composite samples were coated with salivary film, which promotes oral bacteria binding to salivary receptors [67].

Low volumetric shrinkage, good reactivity and mechanical properties are among the main features that justify the use of bisphenol A-glycidyl methacrylate (Bis-GMA) as the most common monomer for dental composite formulation [68]. However, in resin-based dental materials, Bis-GMA is generally mixed with other monomers, such as TEG-DMA and EGDMA, in order to optimize its low viscosity [69,70,71,72,73,74]. In recent years, concerns have been raised as a result of the observed estrogenic action of bisphenol A (BPA) [75] following the salivary hydrolysis of Bis-GMA-based resins [76]. Thus, Bis-GMA free dental resin composites have been introduced, replacing Bis-GMA with different monomers that do not release BPA, such as urethane-dimethacrylate (UDMA). Bis-GMA replacement still needs further investigation [77]. Based on Kim et al., Bis-GMA seems to inhibit the planktonic growth and the viability of *Streptococcus mutans* [78]. Accordingly, Lin et al. [79] recorded a reduced *Streptococcus mutans* activity and biofilm formation in the presence of Bis-GMA. For these reasons, in the present study, a (Bis-GMA)-based (GR) and two (Bis-GMA)-free dental resin composites (VD and BF) were selected. Hansel et al. [40], on the other hand, observed that the release of other co-monomers (such as TEGDMA and EGDMA) could be somehow able to promote the growth of some *Streptococcus* spp. This second mechanism, combined with the well-known reduction in monomers released from heat-cured composites [80], might hypothetically justify the reduced number of sessile *Streptococcus mutans* colonies herein observed on heat-cured materials. Regarding the planktonic CFU count, data recorded on the (Bis-GMA)-free BF were significantly higher than those obtained on the (Bis-GMA)-based GR, both in light-cured and heat-cured subgroups, supporting the potential inhibitory role of Bis-GMA. However, significantly reduced planktonic colonies were observed also with the (Bis-GMA)-free VD, suggesting that such a planktonic *Streptococcus mutans* decrease could also be related to the action of other monomers, or a combination of them [43]. Focusing on the *Streptococcus mutans* sessile colonies, the light-cured VD (Bis-GMA-free) resulted in a significant increase in the CFU/mL count compared to the light-cured BF (Bis-GMA-free as well) and to the light-cured GR (Bis-GMA-based). 

Various heat-curing protocols have been described in the literature, whose times ranged between 10 and 6 h, with different maximum temperature values [81,82,83,84]. A 10 min heat-curing treatment has been shown to be adequate to increase composite properties [85]. Also, in the oral cavity, temperatures can change [86]. Dental materials that resist oral cavity variations up to a temperature of 77.4 °C (i.e., drinking hot fluids) daily can preserve their physical and chemical properties [86]. High temperatures can be responsible of an irreversible deformation and changes in the materials properties [86], justifying the chosen temperature (80 °C) used in our experimental study. Heat-curing protocols promote greater monomer-to-polymer conversion [33,34], limiting monomer leaching [87,88,89]. Brambilla et al. [41] already showed how reduced curing times could be responsible for the increased in vitro colonization of composite surfaces by *Streptococcus mutans.* The authors suggested that this phenomenon could be related to the presence of unpolymerized monomers on the material surface, but the exact biomechanical mechanisms were not fully clarified [90,91]. The results of the present preliminary in vitro study confirmed a general trend towards the reduction for both sessile and planktonic CFUs of heat-cured resin composites.

Regarding the biomass quantification by optical density (OD_570nm_), data recorded in the light-cured BF subgroup were significantly higher than for the same material subjected to a heat-curing protocol. Bacterial colonization is the main agent for caries onset [41]. *Streptococcus mutans* is one of the main promoters of biofilm formation by controlling the matrix development formed by insoluble exopolysaccharides as α1,3-glucans [92,93]. Insoluble exopolysaccharides prevent saliva neutralization [13]. Secondary agents in caries lesions process, such as *Candida albicans* [51], may also promote exopolysaccharide increase and accumulation, enhancing the formation of microcolonies by *Streptococcus mutans* [50,94]. A reduced biofilm biomass is considered less resistant to the effect of a salivary buffer [43]. The long-term outcome of composite restorations is negatively affected by the presence of thick biofilms [95]. Acids and bacteria enzymatic degradation can jeopardize the function and the esthetic longevity of a restoration [96,97,98]. Further composite degradation is related to the *Streptococcus mutans* esterase gene that can catalyze the uncured monomers, causing degradation and marginal bacterial colonization [99], as well as an alteration of surface topography [7]. 

As already mentioned, *Streptococcus mutans* is not the only microorganism involved in oral biofilm formation and decay development. In recent studies, the high prevalence of *Candida albicans* was recovered in bacterial biofilms together with *Streptococcus mutans* [49,50,100,101]. The presence of *Candida albicans* induces the expression of *Streptococcus mutans* glucosyltransferases and genes to withstand environmental stress [50]. Indeed, a greater biofilm complexity makes microorganisms more tolerant to environmental stresses [44]. Further studies should better investigate *Candida albicans* adhesion and proliferation on differently cured composite resins, mainly focusing on the potential interactions between *Streptococcus mutans* and *Candida albicans*. Among the limitations of this study, the use of a mono-species and static bacterial model could limit the generalizability of the obtained findings. Further studies should be carried out to confirm the adhesion properties with multispecies clinical biofilms.

The clinical relevance of the present results should be carefully interpreted, also considering other paramount properties related to the peculiar composition of any different resin-based materials. Whenever a material comes into contact with live tissues and cells they can interact, generating a biological response, and many components of composite resins have shown potential cytotoxic activity [102]. For instance, the residues of free methacrylate monomers following polymerization may trigger the production of prostaglandin E2, cyclooxygenase 2 (COX2) and an increase in interleukin-1B (IL-1B), IL-6 and nitric oxide (NO) [103,104,105]. Residual monomers may be eluted after composite placement [106,107] following both chemical and physical degradation over time [108].

In the present research, composites were treated using already known and well referenced methodologies. Nevertheless, the improvement of the study is related to the observed effect of the different curing treatments on the interaction between the composite disks and *Streptococcus mutans*.

The results obtained in this preliminary in vitro study highlight further potential benefits from an effective resin composite heat-curing cycle. Besides the already known advantages in terms of mechanical properties such as microhardness, flexural strength, wear resistance, tensile strength, fracture toughness and color stability [35,36,37,38], the present results highlighted the following:

- The intrinsic ability of *Streptococcus mutans* to proliferate seems impaired in the presence of composites subjected to additional heat-curing protocols, with a general trend towards a reduction in both sessile and planktonic CFUs;
- Where there is an inability to reduce sessile CFUs, heat-curing still seems to inhibit the production of biofilm glycoproteins.

From a clinical perspective, a reduced adhesion, proliferation and/or matrix production by *Streptococcus mutans*, achieved as a result of additional heat-curing polymerization protocols, could significantly decrease its pathogenicity, thereby reducing the risk of secondary caries. A significant role seems also to be played by the filler particle size, with small-sized fillers leading to reduced *Streptococcus mutans* proliferation.

## Figures and Tables

**Table 1:** Summary of experimental groups and materials employed in the study.

Experimental Group	Curing Protocol	Material	Manufacturer	Lot Number	Composition
GR	Light-curing	GrandioSO Shade A2 (Nanohybrid)	Voco GmbH, Cuxhaven, Germany	2213772	89% fillers (1 μm glass filler); 20–40 nm (silicon dioxide filler); pigments (iron oxide, titanium dioxide). Bis-GMA, Bis-EMA, TEGDMA
	Heat-curing				
VD	Light-curing	Venus Diamond Shade A2 (Nanohybrid)	Kulzer GmbH, Hanau, Germany	K010201	64% fillers (5 nm–20 μm), barium aluminum fluoride glass (Ø 0.7 max. <2 μm), discrete nanoparticles. TCD-DI-HEA, UDMA.
	Heat-curing				
BF	Light-curing	Enamel Plus HRi Biofuntion Shade BF2 (Nanohybrid)	Micerium SpA, Avegno, Italy	2023000990	74% fillers (0.005 µm-0.05 µm silicon dioxide), (0.2–3.0 µm glassy filler). UDMA, Tricyclodecane dimethanol dimethacrylate.
	Heat-curing				

For all light-cured resin composites, a curing of 20 s on both specimen sides with a light-emitting diode curing unit (Celalux 3, VOCO, Cuxhaven, Germany)—output 1300 mW/cm^2^—was realized. For all heat-cured resin composites, after the light-curing process, a subsequent and further polymerization of 10 min in a heat-curing unit (LaborLux, Micerium, Avegno, Genova, Italy) at 80 °C was realized.

**Table 2:** Means (and standard deviations) for SM planktonic CFU count (CFU/mL), sessile CFU count (CFU/mL) and biomass quantification by optical density (OD_570nm_).

***-Planktonic CFU Count (CFU/mL)***			
	**Material**		
**Curing protocol**	**GR**	**VD**	**BF**
Light-cured	7.23 × 10^6 c^_1_ (8.05 × 10^5^)	2.14 × 10^7 b^_1_ (4.03 × 10^6^)	4.40 ×1 0^7 a^_1_ (6.13 × 10^6^)
Heat-cured	4.89 × 10^6 b^_1_ (7.65 × 10^5^)	4.95 × 10^6 b^_2_ (9.19 × 10^5^)	2.80 × 10^7 a^_2_ (4.33 × 10^6^)
***-Sessile CFU Count (CFU/mL)***			
	**Material**		
**Curing protocol**	**GR**	**VD**	**BF**
Light-cured	7.49 × 10^6 b^_1_ (7.88 × 10^5^)	2.93 × 10^7 a^_1_ (6.31 × 10^6^)	6.71 × 10^6 b^_1_ (8.55 × 10^5^)
Heat-cured	3.97 × 10^6 a^_2_ (8.70 × 10^5^)	6.07 × 10^6 a^_2_ (8.19 × 10^5^)	6.38 × 10^6 a^_1_ (4.90 × 10^5^)
***-Biomass Quantification by Optical Density (OD_570nm_)***			
	**Material**		
**Curing protocol**	**GR**	**VD**	**BF**
Light-cured	0.1325 ^b^_1_ (0.0207)	0.1457 ^b^_1_ (0.0376)	0.4280 ^a^_1_ (0.0907)
Heat-cured	0.1464 ^a^_1_ (0.0287)	0.1731 ^a^_1_ (0.0241)	0.1931 ^a^_2_ (0.0490)

Different superscript letters indicate a statistically significant difference between the levels of “Material” factor. The superscript letter “a” indicates highest values. The superscript letter “b” indicates higher values than “c”. The same numbers in subscript indicate no significant differences among the levels of “Curing protocol” factor.

## Data Availability

The data presented in this study are available on request from the corresponding author.
